# Robustness of Voltage-induced Magnetocapacitance

**DOI:** 10.1038/s41598-018-33065-y

**Published:** 2018-10-02

**Authors:** Hideo Kaiju, Takahiro Misawa, Taro Nagahama, Takashi Komine, Osamu Kitakami, Masaya Fujioka, Junji Nishii, Gang Xiao

**Affiliations:** 1 Research Institute for Electronic Science, Hokkaido University, Sapporo, Hokkaido 001-0020 Japan; 2 Graduate School of Engineering, Hokkaido University, Sapporo, Hokkaido 060-8628 Japan; 3 Graduate School of Science and Engineering, Ibaraki University, Hitachi, Ibaraki 316-8511 Japan; 4 Institute of Multidisciplinary Research for Advanced Materials, Tohoku University, Sendai, Miyagi 980-8577 Japan; 5 Department of Physics, Brown University, Providence, RI 02912 USA

## Abstract

One of the most important achievements in the field of spintronics is the development of magnetic tunnel junctions (MTJs). MTJs exhibit a large tunneling magnetoresistance (TMR). However, TMR is strongly dependent on biasing voltage, generally, decreasing with applying bias. The rapid decay of TMR was a major deficiency of MTJs. Here we report a new phenomenon at room temperature, in which the tunneling magnetocapacitance (TMC) *increases* with biasing voltage in an MTJ system based on Co_40_Fe_40_B_20_/MgO/Co_40_Fe_40_B_20_. We have observed a maximum TMC value of 102% under appropriate biasing, which is the largest voltage-induced TMC effect ever reported for MTJs. We have found excellent agreement between theory and experiment for the bipolar biasing regions using Debye-Fröhlich model combined with quartic barrier approximation and spin-dependent drift-diffusion model. Based on our calculation, we predict that the voltage-induced TMC ratio could reach 1100% in MTJs with a corresponding TMR value of 604%. Our work has provided a new understanding on the voltage-induced AC spin-dependent transport in MTJs. The results reported here may open a novel pathway for spintronics applications, e.g., non-volatile memories and spin logic circuits.

## Introduction

A new class of electronic devices based on the spin degrees of freedom has been extensively studied and it has given rise to the field of spintronics^1–7^. One of the most important achievements in this field is the development of magnetic tunnel junctions (MTJs), which has enabled the observation of a large tunneling magnetoresistance (TMR)^3,4,8–12^. Practical applications relying on this TMR effect range from data read heads in hard disk drives to highly sensitive magnetic sensors. The magnetic states of MTJs can also be manipulated by the current induced spin-transfer torque (STT) effect^13–17^. More applications have become possible, e.g., magnetic random access memories (MRAMs)^18–21^, logic-in-memory circuits^22,23^ and random number (RN) generators^24,25^. More recently, neuromorphic computing using spin-torque MTJ oscillators has been developed^26^. Thus, MTJs can serve as one of the most crucial building blocks of spintronics-based computing.

It is well known that TMR is strongly dependent on biasing voltage, generally, decreasing with applying bias^27–30^. One major challenge in MTJ research is to find ways to increase the half biasing voltage, *V*_1/2_, at which the total TMR ratio near zero biasing is halved. For MgO-based MTJs^3^, *V*_1/2_ is approximately 1 V. Normal electrical operations will always require certain applying biasing voltage, which renders TMR less optimal. Extensive efforts have been made to enhance *V*_1/2_, but progress has come very slowly. Recently, it has been demonstrated that *V*_1/2_ depends strongly on the interfacial structures between the ferromagnetic (FM) and the insulator layer. A high *V*_1/2_ can be obtained in MTJs with i) few misfit dislocations near the interfaces, ii) a small lattice strain inside the insulator or iii) well-defined sharp interfaces^31–35^. The value of *V*_1/2_ in lattice-matched Fe/spinel MgAl_2_O_4_/Fe(001) MTJs reaches +1.0 V (−1.3 V) for the positive (negative) bias^31^. In other studies, relatively high *V*_1/2_ values, ranging from 0.5 to 1.0 V, have also been reported in fully epitaxial Fe/MgO/GaO_*x*_/Fe^32^, NiFe(111)/AlO_*x*_/CoFe^33^, Fe(211)/AlO_*x*_/CoFe^34^ and epitaxial-Fe_4_N/MgO/CoFeB MTJs^35^.

Another approach to obtain a high *V*_1/2_ is to explore organic spin valves (OSVs), where carbon-based molecules are used between FM electrodes. The value of *V*_1/2_ for C_60_- and carbon nanotubes-based OSVs is approximately 0.2 V at 5 K^36^. In OSVs using tris(8-hydroxyquinolinate) aluminum (Alq_3_) or rubrene, the *V*_1/2_ has been reported to be 0.1−0.2 V at room temperature^37–39^. Interestingly, graphene-based OSVs show the robustness of spin polarization in a non-local scheme^40^. The normalized spin polarization reaches up to 0.8 at 1.2 V, which is inferior to MgO-based MTJs. Overall, efforts in increasing *V*_1/2_ have not been successful in MTJs or other spintronic devices.

Complementary to TMR effect, the tunneling magnetocapacitance (TMC) is also inherent to the capacitive MTJ structure^41–44^. Recently TMC has been increasingly studied due to their fascinating spin phenomena, such as spin capacitance^45–48^, frequency-dependent spin transport^42,43,49^ and potential applications^41,50^. Though TMC and TMR share some similar origins and are correlated, TMC is unique in many aspects. For example, TMC is peaked at a specific frequency^43^, but is weakly dependent on the temperature^44^. Furthermore, *V*_1/2_ of TMC is higher than that of TMR^44,47^. The highest *V*_1/2_ of TMC in MgO-based MTJs is 0.7 V, which is almost twice as high as that of TMR in the same device^47^. Therefore, TMC is more robust against biasing than TMR, and the mechanism of this robustness remains unclear. It is beneficial to understand this mechanism as the TMC effect may lead to more spintronics applications as well as insight into fundamental spintronic behavior.

In this work we report a new phenomenon at room temperature in which the TMC *increases* with applying biasing voltage in an MTJ system based on Co_40_Fe_40_B_20_/MgO/Co_40_Fe_40_B_20_. This result means an unprecedented enhancement of *V*_1/2_ with regard to TMC. We have observed a maximum TMC value of 102% under biasing, which is the largest voltage-induced TMC effect ever reported for MTJs. There is an excellent agreement between theory and experiment for the TMC in the bipolar bias regions using Debye-Fröhlich (DF) model combined with quartic barrier approximation (QBA) and spin-dependent drift-diffusion (SDD) model. Based on our calculations, we predict that the voltage-induced TMC ratio could reach 1100% in MTJs with a corresponding TMR ratio of 604%.

## Results and Discussion

### Device structure and TMR and TMC under no bias voltage

**Figure 1:**
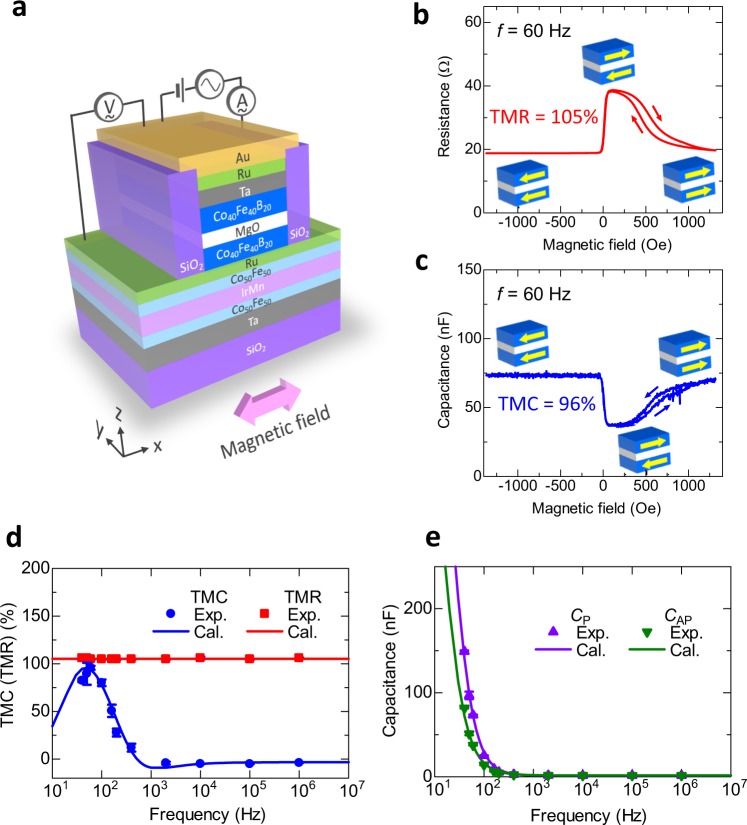
Device structure and TMR and TMC under no bias voltage. (**a**) Schematic of an MgO-based MTJ, with the structure: SiO_2_/Ta(5 nm)/Co_50_Fe_50_(2 nm)/IrMn(15 nm)/Co_50_Fe_50_(2 nm)/Ru(0.8 nm)/Co_40_Fe_40_B_20_(3 nm)/MgO(2 nm)/Co_40_Fe_40_B_20_(3 nm)/contact layer. The measurement set-up for TMC and TMR is also shown. The magnetic field is applied along the magnetic easy-axis direction of Co_40_Fe_40_B_20_ layers. (**b**) TMR and (**c**) TMC curves of an MgO-based MTJ. The frequency is 60 Hz and the DC voltage is 0 V. Frequency dependence of (**d**) TMC and TMR and (**e**) the capacitance *C*_P(AP)_in the P(AP) configuration. The solid plots represent the experimental data and the solid lines represent the calculation results obtained by Debye-Fröhlich model, described by Eq. (2).

Figure 1a shows the device structure prepared by a magnetron sputtering system with a base pressure of 2 × 10^−8^ Torr, with the following layer sequence: SiO_2_/Ta(5)/Co_50_Fe_50_(2)/IrMn(15)/Co_50_Fe_50_(2)/Ru(0.8)/Co_40_Fe_40_B_20_(3)/MgO(2)/Co_40_Fe_40_B_20_(3)/contact layer (thickness in nm). Details of the device fabrication procedure are described in the Experimental Section. Using standard photolithography, we have patterned the multilayer MTJ stacks into a junction area of 1800 μm^2^ with an elliptical shape with Ar ion-milling and SiO_2_ insulation overlayer. The frequency characteristics and the bias voltage dependence of the TMR and TMC for MTJs were measured by an AC four-probe method at room temperature. The AC voltage was set to 2.6 mV_rms_. The magnetic field was applied along the magnetic easy-axis direction to 1.4 kOe.

Figure 1b,c show the TMR and TMC curves at 60 Hz. The DC applied voltage is 0 V. Clear TMR and TMC effects are observed, i.e., *R*_P_ < *R*_AP_ and *C*_P_ > *C*_AP_. Large TMR and TMC ratios of approximately 100% are obtained at room temperature. Figure 1d,e shows the frequency characteristics of TMC, TMR and *C*_P(AP)_. We calculated the frequency characteristics of the TMC and *C*_P(AP)_ using the DF model^43,51^. Based on the model, the capacitance ${C}_{{\rm{P}}({\rm{AP}})}^{{\rm{DF}}}({f})$ as a function of frequency *f* for the P(AP) configuration in MTJs can be expressed by

1 $${C}_{{\rm{P}}({\rm{AP}})}^{{\rm{DF}}}(f)={\rm{Re}}[{C}_{\infty ,{\rm{P}}({\rm{AP}})}+\frac{{C}_{0,{\rm{P}}({\rm{AP}})}-{C}_{\infty ,{\rm{P}}({\rm{AP}})}}{1+{(i2\pi f{\tau }_{{\rm{P}}({\rm{AP}})})}^{{\beta }_{{\rm{P}}({\rm{AP}})}}}],$$

where *C*_∞,P(AP)_ and *C*_0,P(AP)_ are the high-frequency and static capacitances, *τ*_P(AP)_ is the relaxation time of electric dipoles, consisting of electrons and holes, formed near the interface between the FM layer and insulator, and *β*_P(AP)_ is the exponent showing the distribution of the relaxation time, respectively, for the P(AP) configuration. After a straightforward calculation of Eq. (1), we can obtain

2 $${C}_{{\rm{P}}({\rm{A}}{\rm{P}})}^{{\rm{D}}{\rm{F}}}(f)={C}_{{\rm{\infty }},{\rm{P}}({\rm{A}}{\rm{P}})}+\frac{{C}_{0,{\rm{P}}({\rm{A}}{\rm{P}})}-{C}_{{\rm{\infty }},{\rm{P}}({\rm{A}}{\rm{P}})}}{2}[1-\frac{\sinh \,[{\beta }_{{\rm{P}}({\rm{A}}{\rm{P}})}\,{\rm{l}}{\rm{n}}(2\pi \,f{\tau }_{{\rm{P}}({\rm{A}}{\rm{P}})})]}{\cosh \,[{\beta }_{{\rm{P}}({\rm{A}}{\rm{P}})}\,{\rm{l}}{\rm{n}}(2\pi \,f{\tau }_{{\rm{P}}({\rm{A}}{\rm{P}})})]+\,\cos ({\beta }_{{\rm{P}}({\rm{A}}{\rm{P}})}\pi /2)}].$$

The relation between *τ*_P_ and τ_AP_ in FM/insulator/FM is given by ${\tau }_{{\rm{AP}}}={\tau }_{{\rm{P}}}(1+{P}_{{\rm{TMC}}}^{2})/(1-{P}_{{\rm{TMC}}}^{2})$, where *P*_TMC_ is the spin polarization inside the FM layer^43,51^. Using these formula, we can calculate the frequency characteristics of the TMC ratio, defined by ${\rm{TMC}}({f})=({C}_{{\rm{P}}}^{{\rm{DF}}}({f})-{C}_{{\rm{AP}}}^{{\rm{DF}}}({f}))/{C}_{{\rm{AP}}}^{{\rm{DF}}}({f})$. From Fig. 1d,e, it is evident that the theoretical results of the TMC and *C*_P(AP)_ fit the experimental data very well. The calculation was based on the following parameters: *C*_∞,P(AP)_ = 1.45 (1.5) nF, *C*_0,P(AP)_ = 630 (590) nF, *β*_P(AP)_ = 0.998 (0.977), *τ*_P_ = 0.0071 s and *P*_TMC_ = 0.46. As for the magnetoresistance, we calculated the TMR ratio using the Julliere formula^52^, assuming a spin polarization *P*_TMR_ of 0.59. The difference between *P*_TMC_ and *P*_TMR_ is attributed to the different penetration lengths of spin-dependent carriers (inside Co_40_Fe_40_B_20_) contributing to TMC and TMR. Our previous paper reveals that the penetration length (λ_TMC_) of TMC is longer than that (λ_TMR_) of TMR^43^. Here, as is well known, the spin polarization of surface atoms in the FM layer or the interfacial FM atoms between FM/insulator is higher than that of inner atoms from the surface/interface due to the two-dimensional surface/interface effect^53–55^. This picture can be applied to our MTJ system. Namely, the spin polarization (*P*_inter_) of the first interfacial atoms in Co_40_Fe_40_B_20_ layers is considered to be higher than that (*P*_inner_) of inner atoms from the Co_40_Fe_40_B_20_/MgO interface (See Fig. S1). In fact, according to the first-principles calculation using the Vienna *Ab Initio* Simulation Package (VASP), the spin polarization at the Co terminated interface is higher than that of the inner Co and Fe atoms in CoFeB/MgO system^56^. From these spin-polarization behavior, it is found that *P*_TMC_ is low for a long λ_TMC_, and *P*_TMR_ is high for a short λ_TMR_. According to the above fitting results, *P*_TMC_ is 0.46, and *P*_TMR_ is 0.59. The spin polarization *P*_TMR_ of 0.59 is in good agreement with the experimental value (0.57–0.65) of CoFeB alloy obtained by point-contact Andreev reflection^57^. Also, we herein note that a negative TMC, i.e., *C*_P_ < *C*_AP_ , is observed in the high-frequency region. This can be understood from the fitting results, i.e., *C*_∞,P(AP)_ = 1.45 (1.5) nF, as well as the experimental results shown in Fig. 1d. This is due to the appearance of the spin capacitance originated from the accumulation of spin-polarized carriers at the two FM/insulator interfaces. The detailed results and discussion are described in the Supplementary Information section. The excellent agreement between theory and experiment reveals that the TMC shows a maximum value of 96% at 60 Hz. Hence, we will use operation frequency of 60 Hz to study the bias dependence of TMC effect. Here, it is noted that we reported a high TMC of 155% in our previous paper^43^. This result was obtained near zero bias voltage. In contrast, it is found that MTJs showing such high TMC tend to bring about the breakdown under the bias voltage. MTJs showing about 100% do not break down under the bias voltage, and the reproducibility is also good. From this reason, we have studied the bias dependence of TMC using MTJs with a TMC of about 100%.

### Voltage-induced TMC and TMR

**Figure 2:**
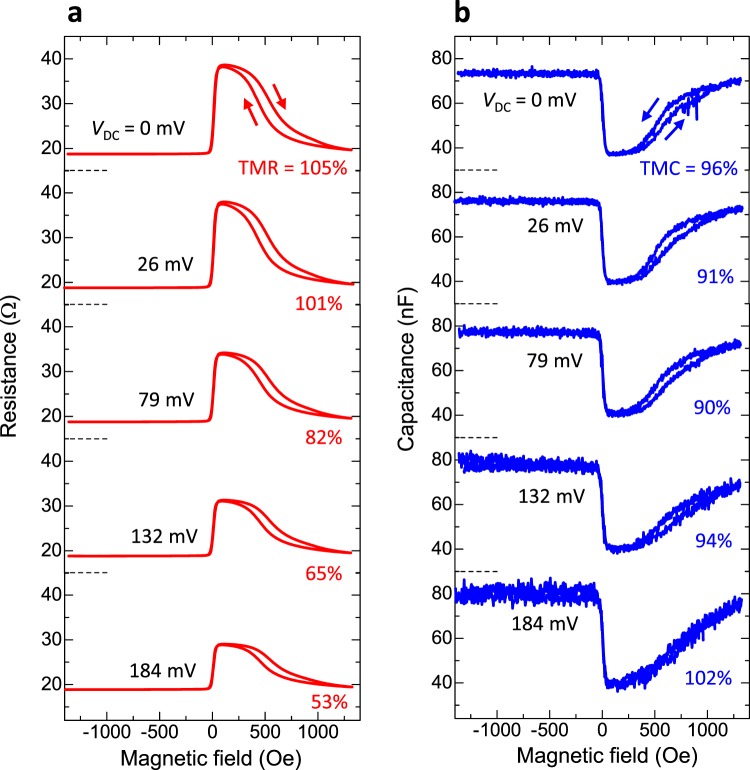
Bias dependence of TMR and TMC. (**a**) TMR and (**b**) TMC curves at 60 Hz in an MgO-based MTJ for positive DC voltages of 0, 26, 79, 132 and 184 mV. The AC voltage is 2.6 mV_rms_.

Figure 2 shows the bias dependence of TMC and TMR curves at 60 Hz. The TMC ratio under the applied bias voltage *V*_DC_ at the frequency *f* is defined by $${\rm{TMC}}({f},{V}_{{\rm{DC}}})$$$$=({C}_{{\rm{P}}}(f,{V}_{{\rm{DC}}})-{C}_{{\rm{AP}}}(f,{V}_{{\rm{DC}}}))/{C}_{{\rm{AP}}}(f,{V}_{{\rm{DC}}})$$. Interestingly, the TMC increases from 96% to 102% as the magnitude of bias voltage increases from 0 to 184 mV, whereas TMR rapidly decreases from 105% to 53%. As described earlier, the enhancement of $${V}_{1/2}^{{\rm{TMR}}}$$ is an important issue in the development of high-performance TMR devices. The typical value of $${V}_{1/2}^{{\rm{TMR}}}$$ is 0.1–1 V in MgO-based MTJs^3,30,58,59^. In our devices, $${V}_{1/2}^{{\rm{TMR}}}$$ is 184 mV. In contrast, since the TMC does not decrease with the increasing bias voltage, the value of $${V}_{1/2}^{{\rm{TMC}}}$$ cannot be defined. In fact, TMC remain robust against bias voltage which weakens the corresponding TMR quickly. Both the large TMC and its robustness are highly beneficial to spintronics applications associated with the TMC effect.

### Modeling of voltage-induced TMC

**Figure 3:**
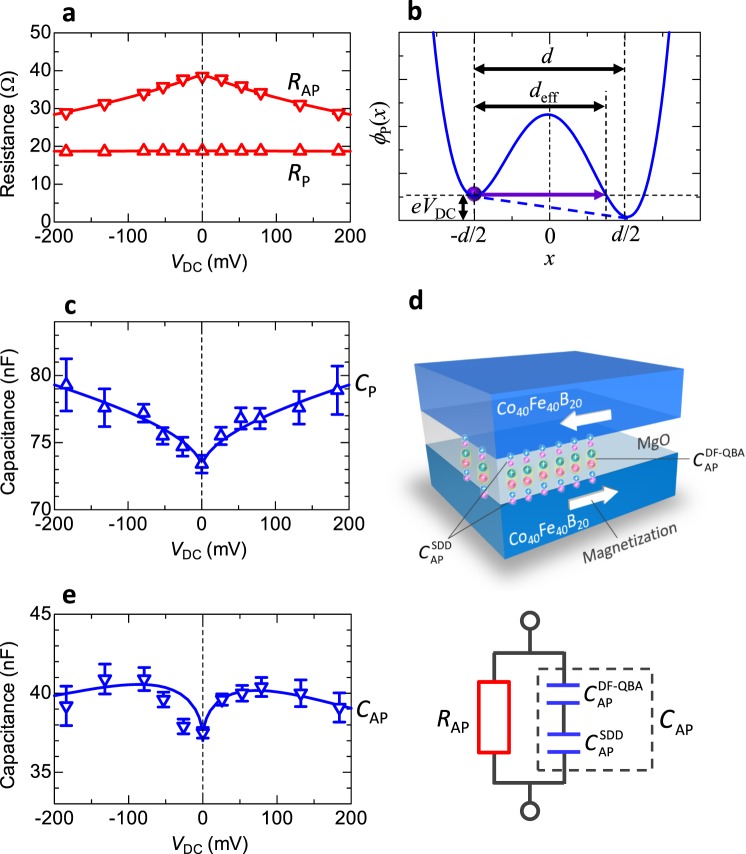
Modeling of the voltage-induced TMC. DC voltage dependence of (**a**) the resistance *R*_P(AP)_ in the P(AP) configuration. The calculation of *R*_P(AP)_ is performed using Zhang’s theory. (**b**) Schematic of the electric potential profile of MTJs under DC voltage. The QBA is used to calculate the effective barrier thickness, which contributes to the measured capacitance. DC voltage dependence of (**c**) the capacitance *C*_P_ in the P configuration. The *C*_P_ is calculated using DF model under the QBA, described by Eq. (3). (**d**) Schematic of charge accumulation, contributing to ${C}_{{\rm{AP}}}^{{\rm{DF}}-{\rm{QBA}}}(f{,}\,{{V}}_{{\rm{DC}}})$ and ${C}_{{\rm{AP}}}^{{\rm{SDD}}}(f{,}\,{{V}}_{{\rm{DC}}})$, and the equivalent circuit in the AP configuration. ${C}_{{\rm{AP}}}^{{\rm{DF}}-{\rm{QBA}}}(f{,}\,{{V}}_{{\rm{DC}}})$ is described by the DF model combined with QBA under the DC voltage *V*_DC_ at the frequency *f* [Eq. (6)]. ${C}_{{\rm{AP}}}^{{\rm{SDD}}}(f{,}\,{{V}}_{{\rm{DC}}})$ is obtained from the SDD model [Eq. (4)]. The equivalent circuit of the MTJ is modeled by the *RC* parallel network, consisting of the resistance *R*_AP_(*V*_DC_) and capacitance *C*_AP_(*f*, *V*_DC_). DC voltage dependence of (**e**) the capacitance *C*_AP_ in the AP configuration.

Figure 3 shows the bias voltage dependence of *R*_P(AP)_, *C*_P(AP)_ and the modeling of the voltage-induced TMC. According to Zhang’s theory^27^, the tunnel conductance in FM/insulator/FM strongly depends on the bias voltage, especially within the order of a few hundred millivolts, due to hot electrons producing spin excitations. The conductance *G*_P(AP)_ (*V*_DC_) at the bias voltage *V*_DC_ in the P(AP) configuration can be expressed by ${G}_{{\rm{P}}({\rm{AP}})}({V}_{{\rm{DC}}})={G}_{{\rm{P}}({\rm{AP}})}^{0}({\rm{1}}+{\gamma }_{{\rm{P}}({\rm{AP}})}{V}_{{\rm{DC}}})$, where ${G}_{{\rm{P}}({\rm{AP}})}^{0}$ is the conductance at zero bias in the P(AP) configuration and *γ*_P(AP)_ is a parameter determined by the Curie temperatures of FM, the density of states (DOS) of itinerant electrons in FM, direct and spin-dependent transfers and spin quantum number within the framework of the transfer Hamiltonian. The resistance *R*_P(AP)_(*V*_DC_) can be calculated from ${R}_{{\rm{P}}({\rm{AP}})}({V}_{{\rm{DC}}})=1/{G}_{{\rm{P}}({\rm{AP}})}({V}_{{\rm{DC}}})$. As shown in Fig. 3a, the experimental data of *R*_P(AP)_(*V*_DC_) are in good agreement with the calculation using Zhang’s model, where *γ*_P(AP)_ is set to 0.0188 (1.87). This means that Zhang’s model is effective for explaining the bias dependence of TMR under both AC and DC mode.

The calculation of *C*_P_(*f*, *V*_DC_) is performed using the DF model combined with QBA. In the QBA, the potential profile of the barrier is assumed to be a quartic function, which is considered to be a good approximation to describe an AC tunneling transport^51^. The potential function ${\varphi }_{{\rm{P}}}(\eta )$ under the bias voltage *V*_DC_ in the P configuration can be expressed by ${\varphi }_{{\rm{P}}}(\eta )=16{\varphi }_{0,{\rm{P}}}{\eta }^{4}-8{\varphi }_{0,{\rm{P}}}{\eta }^{2}+{\varphi }_{0,{\rm{P}}}+e{V}_{{\rm{D}}{\rm{C}}}(1-2\eta )/2$, where ${\rm{\eta }}=x/d$ is the reduced spatial variable, *x* is the distance from the center of the barrier, *d* and *ϕ*_0,P_ are the barrier thickness and height in the absence of the bias voltage, respectively, and *e* is the electron charge. The potential profile is depicted in Fig. 3b. The solution of ${\varphi }_{{\rm{P}}}(\eta )=e{{V}}_{{\rm{DC}}}$ is *η*_1_ = −1/2, *η*_2_, *η*_3_ and *η*_4_ (for *η*_2_ < 0, *η*_3_ and *η*_4_ > 0, *η*_3_ < *η*_4_). Since the values of *η*_2_, *η*_3_ and *η*_4_ can be calculated by the Cardano’s method, the effective barrier thickness *d*_eff_ can be represented by *d*_eff_ = (1/2 + *η*_3_)*d*, where *η*_3_ depends on *V*_DC_ and *ϕ*_0,P_. Therefore, *C*_P_(*f*, *V*_DC_) at the applied DC voltage in the P configuration can be written by

3 $${C}_{{\rm{P}}}(f,{V}_{{\rm{DC}}})=\frac{1}{1/2+{\eta }_{3}({V}_{{\rm{DC}}},{\varphi }_{0,{\rm{P}}})}[{C}_{\infty ,{\rm{P}}}+\frac{{C}_{0,{\rm{P}}}-{C}_{\infty ,{\rm{P}}}}{2}(1-\frac{\sinh \,[{\beta }_{{\rm{P}}}\,\mathrm{ln}(2\pi f{\tau }_{{\rm{P}}})]}{\cosh \,[{\beta }_{{\rm{P}}}\,\mathrm{ln}(2\pi f{\tau }_{{\rm{P}}})]+\,\cos ({\beta }_{{\rm{P}}}\pi /2)})].$$

As shown in Fig. 3c, the measured capacitance *C*_P_(*f*, *V*_DC_) exhibiting a cusp-like behavior, is very well described by Eq. (3), using parameters of *C*_∞,P_ = 1.45 nF, *C*_0,P_ = 630 nF, *β*_P_ = 0.998, *τ*_P_ = 0.0075 s, *f* = 60 Hz and *ϕ*_0,P_ = 2.15 eV. Hence, the QBA is a good approximation for the expression of the potential-barrier profile in MTJs based on Co_40_Fe_40_B_20_/MgO/Co_40_Fe_40_B_20_ in calculating the bias dependence of *C*_P_ under AC field.

The calculation of *C*_AP_(*f*, *V*_DC_) is performed using the DF model combined with QBA and SDD model. Based on the SDD model^60^, the accumulation of minority spins and the depletion of majority spins take place at the interface between the Co_40_Fe_40_B_20_ and MgO layers in the AP configuration. The spin accumulation causes a difference in the chemical potential between majority and minority spins, resulting in a different diffusion length in each spin. The difference in the diffusion length gives rise to the creation of tiny screening charge dipoles, which act as an additional serial capacitance, i.e., spin capacitance. The screening charge density is given by $e{n}_{{\rm{AP}}}({x}_{i})=e{n}_{0,\mathrm{AP}}\,\exp (\,-\,{x}_{i}/\lambda )$, where *x*_*i*_ is the distance from the interface between the Co_40_Fe_40_B_20_ and MgO, *λ* is a characteristic screening length and *en*_0,AP_ is a screening charge density at the interface in the AP configuration. The spin capacitance can be expressed by ${{C}}_{{\rm{AP}}}^{{\rm{SDD}}}({V}_{{\rm{DC}}})={\rm{\Delta }}{Q}_{{\rm{AP}}}/{\rm{\Delta }}{V}_{{\rm{DC}}}$, where Δ*Q*_AP_ is the screening charge for the AP configuration and Δ*V*_DC_ is the electrical potential difference applied in the charging space. Therefore, the spin capacitance can be obtained as ${{C}}_{{\rm{AP}}}^{{\rm{SDD}}}({V}_{{\rm{DC}}})=eS{n}_{{\rm{AP}}}({x}_{i})d{x}_{i}/d{V}_{{\rm{DC}}}({x}_{i})$, where *S* is a junction area and *V*_DC_(*x*_*i*_) is an electrical potential as a function of *x*_*i*_ in the charging space, i.e., ${V}_{{\rm{DC}}}({x}_{i})={V}_{{\rm{eff}}}\,\exp (\,-\,{x}_{i}/\lambda )$. *V*_eff_ is an effective applied voltage, which is expressed by *κV*, where *κ* is an adjustable positive parameter of much smaller than 1.0. Consequently, the spin capacitance can be represented by

4 $${C}_{{\rm{AP}}}^{{\rm{SDD}}}({V}_{{\rm{DC}}})=eS\frac{{n}_{0,{\rm{AP}}}\lambda }{\kappa {V}_{{\rm{DC}}}}.$$

Since this screening charge acts as a serial capacitance, the capacitance *C*_AP_(*f*, *V*_DC_) under the applied DC voltage *V*_DC_ in the AP configuration,

5 $${C}_{{\rm{AP}}}(f,{V}_{{\rm{DC}}})={(\frac{1}{{C}_{{\rm{AP}}}^{{\rm{DF}}-{\rm{QBA}}}(f,{V}_{{\rm{DC}}})}+\frac{1}{{C}_{{\rm{AP}}}^{{\rm{SDD}}}({V}_{{\rm{DC}}})})}^{-1},$$

where the capacitance ${C}_{{\rm{AP}}}^{\mathrm{DF}-\mathrm{QBA}}$ (*f*, *V*_DC_) based on the DF model combined with QBA in the AP configuration is represented by

6 $$\begin{array}{rcl}{C}_{{\rm{AP}}}^{{\rm{DF}}-{\rm{QBA}}}(f,{V}_{{\rm{DC}}}) & = & \tfrac{1}{1\,/\,2+{\eta }_{3}((1-\kappa ){V}_{{\rm{DC}}},{\varphi }_{0,{\rm{AP}}})}\\  &  & \times \,[{C}_{\infty ,{\rm{AP}}}+\tfrac{{C}_{0,{\rm{AP}}}-{C}_{\infty ,{\rm{AP}}}}{2}(1-\tfrac{\sin \,{\rm{h}}[{\beta }_{{\rm{AP}}}\,\mathrm{ln}(2\pi f{\tau }_{{\rm{AP}}})]}{\cos \,{\rm{h}}[{\beta }_{{\rm{AP}}}\,\mathrm{ln}(2\pi f{\tau }_{{\rm{AP}}})]+\,\cos \,({\beta }_{{\rm{AP}}}\pi /2)})].\end{array}$$

The behavior of charge accumulation, contributing to ${{C}}_{{\rm{AP}}}^{\mathrm{DF}-\mathrm{QBA}}$ and ${{C}}_{{\rm{AP}}}^{{\rm{SDD}}}$, is illustrated in Fig. 3d. The bias voltage dependence of the capacitance *C*_AP_ in the AP configuration is shown in Fig. 3e. The capacitance *C*_AP_ increases at around zero bias and then it decreases at higher voltages. This behavior is in good agreement with the results calculated by Eqs (4)−(6) with parameters of *C*_∞,AP_ = 1.5 nF, *C*_0,AP_ = 590 nF, *β*_AP_ = 0.977, *τ*_P_ = 0.0075 s, *P*_TMC_ = 0.46, *f* = 60 Hz, *κ* = 0.1, *S* = 1800 μm^2^ and λ = 0.1 nm. *ϕ*_0,AP_ is 0.144 (0.153) eV and *n*_0,AP_ is 0.94 (0.92) × 10^23^ cm^−3^ for the positive (negative) bias region. The increase of *C*_AP_ near zero bias is attributed to the decrease of the effective barrier thickness *d*_eff_. As seen from the potential profile *ϕ*_P_(*x*) of MTJs shown in Fig. 3b, *d*_eff_ decreases with increasing bias voltage *V*_DC_. This corresponds to the decrease of *η*_3_. According to our calculation, *η*_3_ is 0.5, 0.35 and 0.28 for *V*_DC_ of 0, 50 and 100 mV, respectively. From Eq. (6), it is found that ${{C}}_{{\rm{AP}}}^{{\rm{DF}}-{\rm{QBA}}}(f,{V}_{{\rm{DC}}})$ increases with decreasing *η*_3_. Therefore, *C*_AP_(*f*, *V*_DC_) increases with increasing *V*_DC_. The reduction of *C*_AP_ at higher voltages is attributed to the spin capacitance, described in Eq. (4), i.e., the spin capacitance decreases with increasing *V*_DC_.

**Figure 4:**
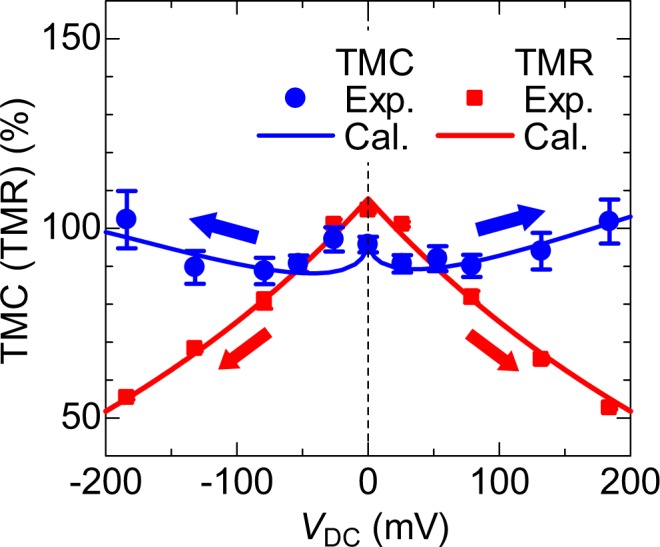
Bias dependence of TMC and TMR ratio. The solid lines represent the calculation results performed using Eqs (3)−(6) for TMC and Zhang formula for TMR. The parameters used in this calculation are described in the main text. The TMC and TMR provide excellent fits to experimental data in bipolar bias regions.

Figure 4 shows the bias-voltage dependence of the TMC and TMR ratios. The calculation of the TMC is performed using Eqs (3–6) for setting the same parameters used in the calculation of *C*_P(AP)_ (*f*, *V*_DC_) in Fig. 3c,e. The TMR is obtained from the Zhang formula. The calculation results of the TMC and TMR provide excellent fits to experimental data in bipolar bias regions. The reduction of TMR can be easily understood from the experimental results of both no significant change of *R*_P_ (*V*_DC_) and the reduction of *R*_AP_ (*V*_DC_) in higher voltages, as shown in Fig. 3a. In particular, the reduction of *R*_AP_ (*V*_DC_) is due to the existence of hot electrons tunneling through the barrier, which brings about the decrease of TMR. The enhancement of TMC can also be clearly understood from the results of both the increase of *C*_P_ (*f*, *V*_DC_) and the reduction of *C*_AP_ (*f*, *V*_DC_) in higher voltages, as shown in Fig. 3c,e. The reduction of *C*_AP_ (*f*, *V*_DC_) is attributed to the emergence of the spin capacitance, which promotes the increase of TMC. Thus, the spin capacitance gives a significant influence on TMC, and it will play an important role on future application.

Though the application of the interesting TMC effect is not well understood at this stage, we venture to discuss the application potential of TMC devices. As can be seen from Fig. 4, TMR is larger than TMC in the low bias region. In considering the application, the important factor in sensing is an electric sensitivity. The electric sensitivity is generally expressed by the signal-to-noise (S/N) ratio, which is defined as 20log*V*_S_/*V*_N_. Here *V*_S_ is a signal voltage and *V*_N_ is a noise voltage. In the case of TMR sensing under the applied voltage *V*_DC_, since *V*_S_ is given by $TMR\cdot {V}_{{\rm{DC}}}$ and *V*_N_ is proportional to $\sqrt{{V}_{{\rm{DC}}}}$, *V*_S_/*V*_N_ is proportional to $TMR\cdot \sqrt{{V}_{{\rm{DC}}}}$. Therefore, the increase of *V*_DC_ is necessary for the improvement of the S/N ratio. In fact, magnetic read heads, sensors, or MRAMs are designed for operation under a bias of a few hundred mV^61^. Additionally, the enhancement of TMR at a higher voltage is of importance. As shown in Fig. 4, the TMR decreases with increasing *V*_DC_, whereas TMC increases at higher voltages. Since *V*_S_/*V*_N_ is proportional to $TMC\cdot \sqrt{{V}_{{\rm{DC}}}}$ in TMC sensing, the TMC is superior to TMR from the viewpoint of the S/N ratio. Furthermore, the impedance *Z* of TMC devices can be reduced in the high frequency region since the impedance of the capacitor is expressed as *Z* = 1/*jωC*, where *ω* is the angular frequency. This means that the noise voltage *V*_N_ could be reduced. The high *V*_S_ and low *V*_N_ lead to the enhancement of the S/N ratio. Also, the AC technique allows for the performance of various functions such as modulation/demodulation, filtering, oscillation and resonance. In fact, magnetoimpedance devices using the AC technique have enabled the application to geomagnetic sensors or positioning sensors of GPS^62–65^. Also, a capacitive magnetic sensing, in which an RC parallel circuit module consisting semiconductor magnetoresistance device and capacitor was used in the feedback loop of a Hartley or Colpitts oscillator, has been proposed and demonstrated by our group^66–68^. In short, TMC devices may pave a new way for various applications to the next generation of magnetic read heads, MRAMs, logic circuits, and highly sensitive magnetic sensors (including geomagnetic sensors, positioning sensors, etc.).

### Prediction of an extremely large voltage-induced TMC

**Figure 5:**
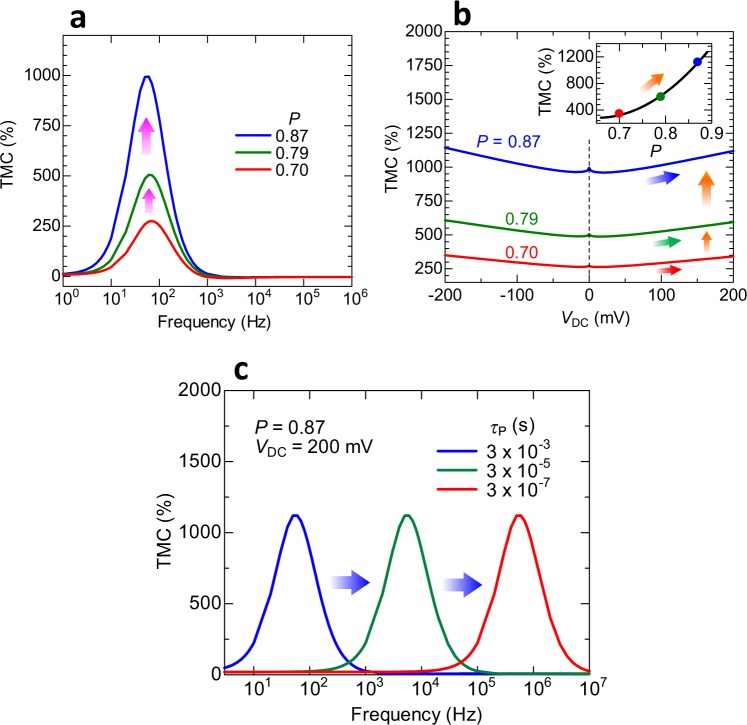
Calculated frequency dependence of extremely large voltage-induced TMC. (**a**) Calculated frequency dependence of the TMC at *V*_DC_ = 0 V in varying *P*. The TMC, showing the maximum value at *f* = 60 Hz, increases from 273% to 995% with increasing *P* from 0.70 to 0.87. (**b**) Calculated DC voltage dependence of the TMC. The calculation result predicts that the voltage-induced TMC could potentially reach 1100% in MTJs with a *P* of 0.87, which is within the realm of high-performance MTJs (See ref.^12^). (**c**) Calculated frequency dependence of the voltage-induced TMC with varying *τ*_P_. The peak position of the TMC is shifted to a high frequency region for a short *τ*_P_.

Finally, the spin polarization dependence of the voltage-induced TMC is calculated for the prediction of an extremely large TMC. Figure 5a,b shows the calculated frequency dependence of the TMC under no bias voltage and the bias dependence of the TMC with varying *P*, respectively. Here, *P*_TMC_ is assumed to be equal to *P*_TMR_, which is denoted by *P*. The assumed maximum value of *P* is 0.87, which is estimated experimentally for high-performance MgO-based MTJs at room temperature^12^. The parameters used in the calculation of the TMC are *C*_∞,P(AP)_ = 1.45 (1.5) nF, *C*_0, P(AP)_ = 184 (172) nF, *β*_P(AP)_ = 0.998 (0.932) and *τ*_P_ = 0.0033 s. As can be seen from Fig. 5a, the TMC ratio at *V*_DC_ = 0 V shows the maximum value at *f* = 60 Hz for the *P* of 0.70, 0.79 and 0.87, respectively, and it increases from 273% to 995% with increasing *P* from 0.70 to 0.87. The maximum TMC of 273%, 505% and 995% are larger than TMR ratios of 192%, 325% and 604%, which are calculated from Julliere formula using *P* = 0.70, 0.79 and 0.87, respectively. The parameters used in the calculation of the bias dependence of TMC are *f* = 60 Hz, *κ* = 0.1, *S* = 1800 μm^2^ and λ = 0.1 nm. The barrier height *ϕ*_0,P_ is 2.15 eV, and *ϕ*_0,AP_ is 0.144 (0.153) eV in the positive (negative) bias voltage. The screening charge densities *n*_0,AP_ are 0.312 (0.306), 0.208 (0.204) and 0.125 (0.123) × 10^23^ cm^−3^ in the positive (negative) bias voltage for the spin polarizations *P* of 0.70, 0.79 and 0.87, respectively. The TMC increases from 995% to 1119% with increasing *V*_DC_ from 0 to 200 mV. Figure 5c shows the calculated frequency dependence of the TMC at *V*_DC_ = 200 mV with varying *τ*_P_. The maximum peak of the TMC is shifted to a high frequency region on the order of MHz for a short *τ*_P_ in the sub-μs scale. According to the DF model, *τ*_p_ can be tuned by changing the oscillation speed of electric dipoles formed near the FM/insulator interfaces; *τ*_p_ is short for a high oscillation speed. From the viewpoint of MTJ device structure, *τ*_p_ can be tuned by changing the thickness of the insulating barrier; *τ*_p_ could shorten in MTJs with a thinner barrier. Although this realization in MTJs is future work, the recent study using FeCo-MgF nanogranular films has demonstrated that the TMC peak is observed at a high frequency of 220 MHz, which corresponds to *τ*_p_ of 0.72 ns^69^. Our calculations predict that a large TMC of over 1100% could be possibly observed using MTJs with a realistic *P* of 0.87. Furthermore, this large TMC can be tuned from low to high frequencies by shortening *τ*_P_.

In summary, we observed a new phenomenon in which the TMC *increases* with the bias voltage in MgO-based MTJs at room temperature. The enhancement of TMC is attributed to the emergence of the spin capacitance in the AP configuration of MTJs. The voltage-induced TMC increases to 102%, which is the largest value ever reported for MTJs. We also found the voltage-induced TMC can be well explained by a newly proposed theoretical calculation using DF model combined with QBA and SDD model. This calculation predicts that the voltage-induced TMC could potentially reach 1100% in MTJs with a corresponding TMR value of 604%. These theoretical and experimental findings provide a deep insight into the voltage-induced AC spin transport in MTJs. We demonstrated that the complementary TMR and TMC effects must be treated on equal footing. The large TMC effect and the associated robustness against biasing may open up new avenues for spintronics applications and electrical modeling.

## Methods

### Preparation of the samples

The MTJs were prepared by using a magnetron sputtering system with a base pressure of 2 × 10^−8^ Torr. The MTJs have the following layer sequence: SiO_2_/Ta(5)/Co_50_Fe_50_(2)/IrMn(15)/Co_50_Fe_50_(2)/Ru(0.8)/Co_40_Fe_40_B_20_(3)/MgO(2)/Co_40_Fe_40_B_20_(3)/contact layer (thickness in nm). We deposited all the metallic layers in DC mode under a sputtering Ar gas pressure of 1.5 mTorr. The MgO layer was deposited with radio frequency (RF) magnetron sputtering at an Ar gas pressure of 1.1 mTorr. Using standard photolithography, we have patterned the multilayer MTJ stacks into a junction area of 1800 μm^2^ with an elliptical shape with Ar ion-milling and SiO_2_ insulation overlayer. Finally, we annealed the MTJs at 310 °C for 4 h in vacuum of 10^−6^ Torr under a uniform magnetic field of 4.5 kOe to define the pinning axis for the Co_40_Fe_40_B_20_ bottom electrode.

### Measurements of the voltage-induced TMC

The frequency characteristics and the bias voltage dependence of the TMR and TMC for MTJs were measured by an AC four-probe method using an Agilent Technologies 4284 A LCR meter at room temperature. The frequency ranged from 50 Hz to 1 MHz and the bipolar bias voltage was applied up to 200 mV. The AC voltage was set to 2.6 mV_rms_. The magnetic field was applied along the magnetic easy-axis direction to 1.4 kOe.

## Electronic supplementary material

Supplementary Information
