# Supplementary material for: Robustness of Voltage-induced Magnetocapacitance
Source: Sci Rep. 2018 Oct 2;8:14709. doi: 10.1038/s41598-018-33065-y (PMC6168469; doi:10.1038/s41598-018-33065-y)
Supplement: Supplementary file 1 — Supplementary Information [file 41598_2018_33065_MOESM1_ESM.docx]

**Robustness of Voltage-induced Magnetocapacitance**

**Supplementary Information**

**Hideo Kaiju^1^, Takahiro Misawa^1^, Taro Nagahama^2^, Takashi Komine^3^,**

**Osamu Kitakami^4^, Masaya Fujioka^1^, Junji Nishii^1^ and Gang Xiao^5^**

**^1^Research Institute for Electronic Science, Hokkaido University, Sapporo, Hokkaido 001-0020, Japan**

**^2^Graduate School of Engineering, Hokkaido University, Sapporo, Hokkaido 060-8628, Japan**

**^3^Faculty of Engineering, Ibaraki University, Hitachi, Ibaraki 316-8511, Japan**

**^4^Institute of Multidisciplinary Research for Advanced Materials, Tohoku University, Sendai, Miyagi 980-8577, Japan**

**^5^Department of Physics, Brown University, Providence, RI 02912, USA**

Correspondence and requests for materials should be addressed to H. K. (email: kaiju@es.hokudai.ac.jp).

**In this Supplementary Information section, we present the relation between the spin polarization and penetration length in TMC and TMR, and detailed results on the frequency characteristics of TMC under no bias voltage.**

**Relation between the spin polarization and penetration length in TMC and TMR.** In TMC, spin-dependent carriers with a penetration length *λ*_TMC_ of a certain width (~nm) contribute to AC transport^1^. This means that TMC is determined by spin polarization of carriers in the FM layers *near* the interface between FM/insulator, as shown in Fig. S1a. In contrast, TMR is determined by spin polarization of carriers in the FM layers *at* the interface between FM/insulator, i.e. the penetration length *λ*_TMR_ is short, typically, an atomic order, as shown in Fig. S1b. As described in the main text, the spin polarization of surface atoms in the FM layer or the interfacial FM atoms between FM/insulator is higher than that of inner atoms from the surface/interface atoms due to the two-dimensional surface/interface effect^2−4^. This picture can be applied to our MTJ system. Namely, as shown in Fig. S1, the spin polarization (*P*_inter_) of the first interfacial atoms in Co_40_Fe_40_B_20_ layers is higher than that (*P*_inner_) of inner atoms from the Co_40_Fe_40_B_20_/MgO interface. In fact, according the first-principles calculation using the Vienna *Ab Initio* Simulation Package (VASP), the spin polarization at the Co terminated interface is higher than that of the inner Co and Fe atoms in CoFeB/MgO system^5^. From these spin-polarization behavior, it is found that *P*_TMC_ is low for a long *λ*_TMC_, and *P*_TMR_ is high for a short *λ*_TMR_.

**a**

**b**


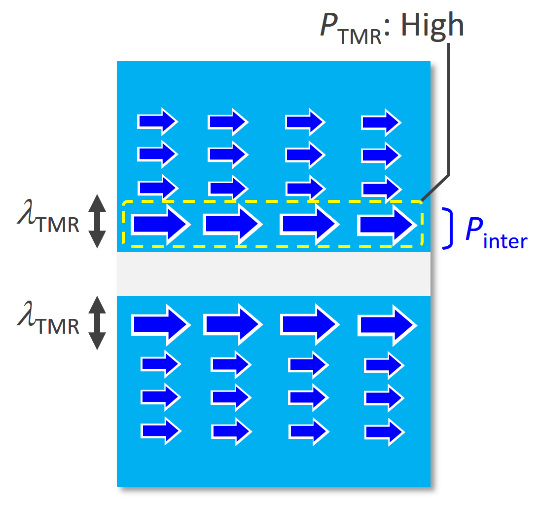
_
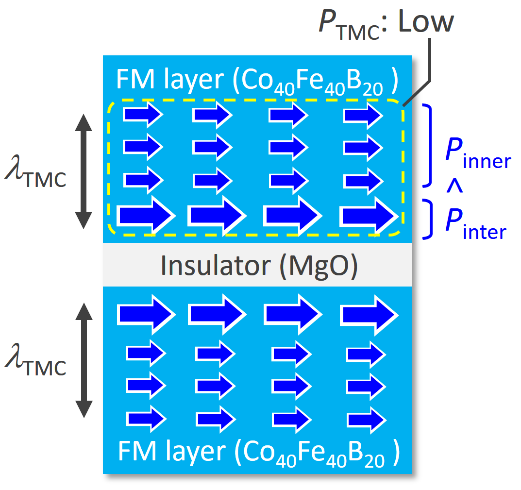
_

**Figure S1 | Relation between the spin polarization and penetration length.** Spin polarization and penetration length of spin-dependent carriers (inside Co_40_Fe_40_B_20_) contributing to (**a**) TMC and (**b**) TMR.

**Frequency characteristics of TMC.** As shown in Fig. S2a, b, the TMC is positive at low frequencies of 50, 60 and 100 Hz, meanwhile it is negative at high frequencies of 10 k, 100 k and 1 MHz; the negative TMC means that *C*_P_ is lower than *C*_AP_. The TMC ratios as a function of frequency have been plotted in Fig. 1d. Fig. S2c shows the TMR and TMC at 1 MHz. The sign of TMR and TMC is opposite to each other, i.e., TMR is positive and TMC is negative. This behavior has also been observed in Co/Al_2_O_3_/Ni_80_Fe_20_ MTJs^6^. The negative TMC is attributed to the presence of spin capacitance at the interface between the FM layer and insulator. TMC is a carrier-mediated phenomenon, in which the carriers are only electrons at high frequencies, and originates from the capacitive accumulation of spin-polarized carriers at the interface^7^. From this physical picture, it is considered that the negative TMC is due to the spin-dependent screening length of the spin-polarized charge accumulation, i.e., the screening length in the P configuration is longer than that in the AP configuration. Finally, we discuss how the spin capacitance is
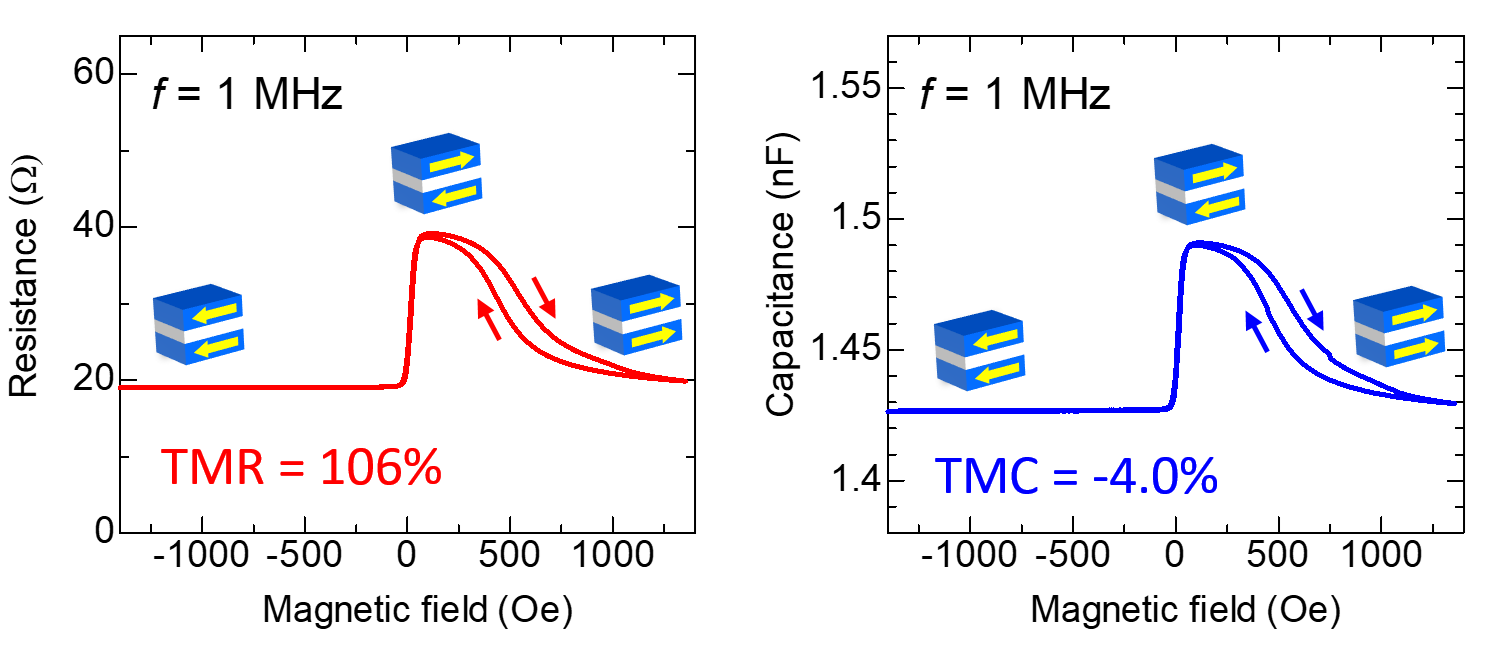

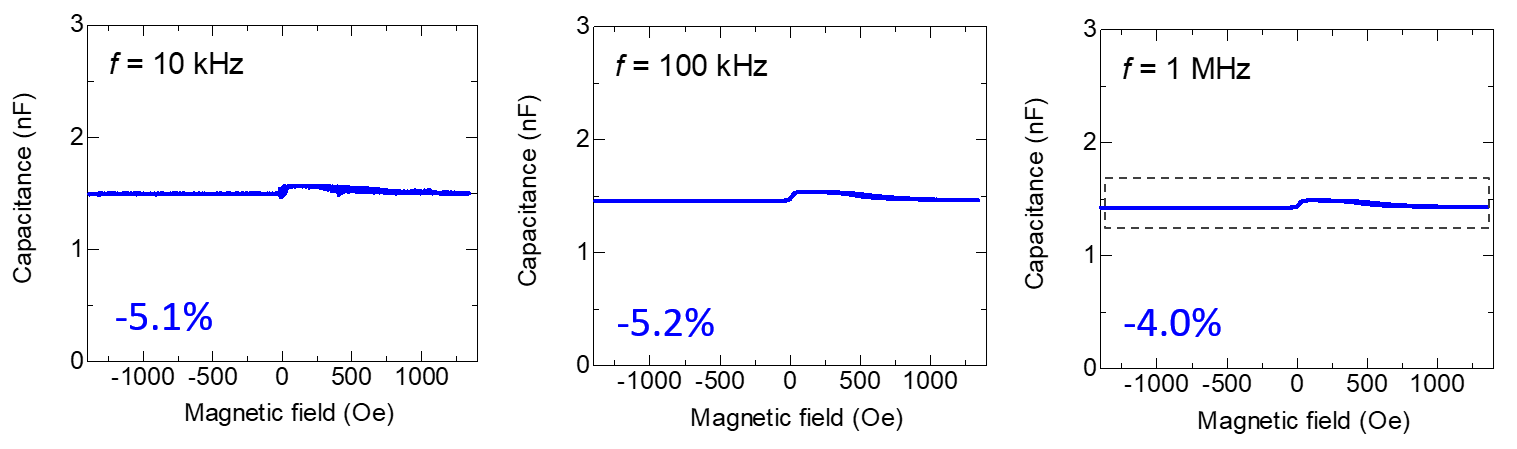

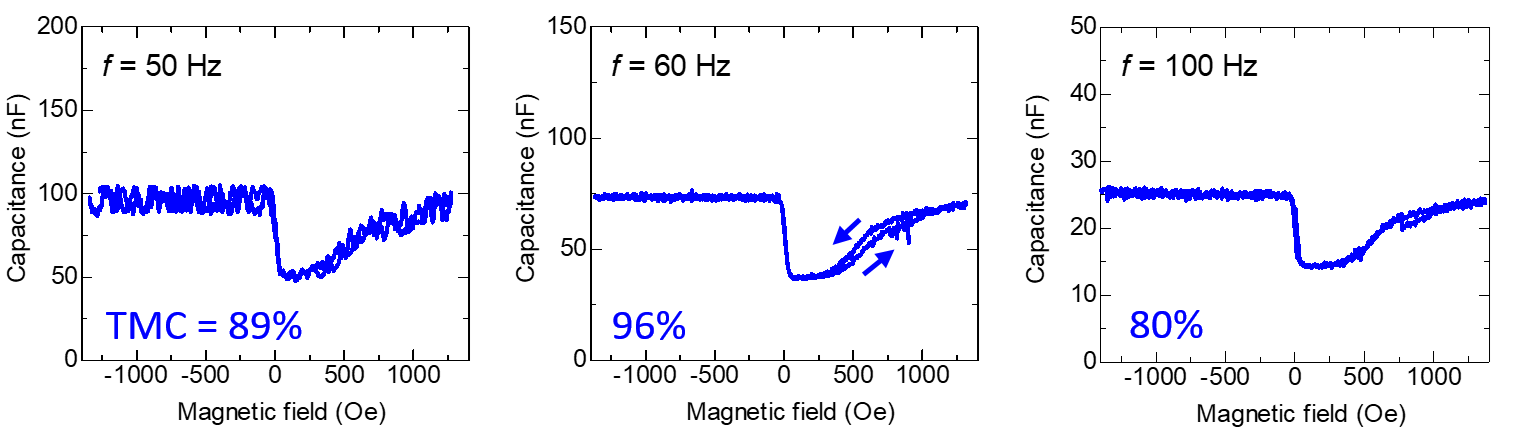
sensitive to certain AC-frequency. As mentioned above, the spin capacitance is observed within the framework of a carrier-mediated phenomenon^7^. In our MTJ system, the carriers are electrons. This means that the spin capacitance is appeared in the entire measured frequency region since the measured frequency is less than 1 MHz in this study. On the other hand, the DF-based capacitance, which originates from the dielectric polarization of the dipoles, formed by electrons and holes near the FM/insulator interfaces, is prominent in the low frequency region, as shown in Fig. 1e. Therefore, the spin capacitance is dominant in the high frequency region, in which the DF-based capacitance approaches zero. According to our data and recently reported results by other groups^6^, the spin capacitance becomes pronounced at higher than ~10 kHz.

**Figure S2 | Frequency characteristics of TMC under no bias voltage.** TMC curves of an MgO-based MTJ at (**a**) low and (**b**) high frequencies. A positive TMC is observed at 50, 60 and 100 Hz, while a negative TMC is observed at 10 k, 100 k and 1 MHz. (**c**) TMR and TMC curves at 1 MHz. TMR and TMC are positive and negative, respectively, at high frequencies.

**b**

**c**

**a**

**Diagram for the TMC calculation.** Figure S3 shows the diagram for TMC calculation, which is beneficial to understand calculation procedure using Eqs. (3)–(6).


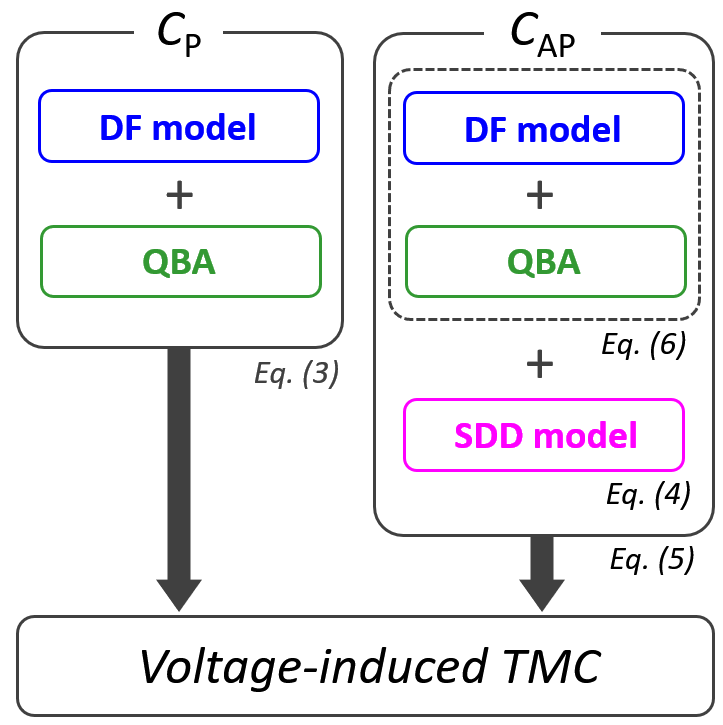


**Figure S3 | Diagram for the TMC calculation.** The voltage-induced TMC is calculated based on this procedure using Eqs. (3)–(6).

**Complementary TMC analysis.** Figure S4 shows experimental data and calculation results on the bias dependence of TMC. The calculation is carried out using Eqs. (3)–(6) with varying *P*_TMC_ and *λ*, respectively. The parameters used in this calculation are described in p.10 and p.11 of the main text. We have found excellent agreement between experiments and calculations performed using *P*_TMC_ = 0.46 and *λ* = 0.1 nm. Here we also note that TMC is sensitive to *P*_TMC_ and *λ*. The sensitivity is not limited to *P*_TMC_ and *λ*. Thus, we conclude that our fitting procedure provides compelling results to explain the experimental data.

**a**

**b**

**Figure S4 | Experimental data and calculation results on the bias dependence of TMC.** The calculation is carried out using Eqs. (3)–(6) with varying (**a**) *P*_TMC_ and (**b**) *λ*, respectively.

**Geometrical capacitance and universal nature of TMC.** In our calculation, we do not take the geometrical capacitance into consideration. Since the junction area is large at 1800 μm^2^, we can neglect the influence of the geometrical capacitance. Under this assumption, there is an excellent agreement between theory and experiment for the bipolar biasing regions using our proposed model, as shown in Figs. 3 and 4.

Finally, we discuss the universal nature of voltage-induced TMC. According to our analysis, the emergence of the spin capacitance leads to the reduction of the capacitance *C*_AP_ in the anti-parallel configuration, which promotes the enhancement of TMC. Therefore, in principle, it is considered that the TMC increases with increasing bias voltage for any tunnel barrier.

**References**

1. Kaiju, H., Takei, M., Misawa, T., Nagahama, T., Nishii, J. & Xiao, G. Large magnetocapacitance effect in magnetic tunnel junctions based on Debye-Fröhlich model. *Appl. Phys. Lett.* **107**, 132405 (2015).
2. Fullerton, E. E., Stoeffler, D., Ounadjela, K., Heinrich, B., Celinski, Z. & Bland, J. A. C. Structure and magnetism of epitaxially strained Pd(001) films on Fe(001): Experiment and theory. *Phys. Rev. B* **51**, 6364−6378 (1995).
3. Fujii, Y., Komine, T., Kai, T. & Shiiki, K. A theoretical study of interfacial structure of Co/Cu and Co/Pd multilayers. *J. Phys.: Condens. Matter* **11**, 9601−9609 (1999).
4. Shiiki, K., Sakaguchi, N. & Kaiju, H. Effect of unoxidized residual Al at the boundary of Co/Al-oxide/Co junction on TMR estimated by LMTO band calculation. *Thin Solid Films* **505**, 64−66 (2006).
5. Burton, J. D., Jaswal, S. S., Tsymbal, E. Y., Mryasov, O. N. & Heinonen, O. G. Atomic and electronic structure of the CoFeB/MgO interface from first principles. *Appl. Phys. Lett.* **89**, 142507 (2006)**.**
6. Parui, S., Ribeiro, M., Atxabal, A., Bedoya-Pinto, A., Sun, X., Llopis, R., Casanova, F. & Hueso L. E. Frequency driven inversion of tunnel magnetoimpedance and observation of positive tunnel magnetocapacitance in magnetic tunnel junctions. *Appl. Phys. Lett.* **109**, 052401 (2016).
7. Rondinelli, J. M., Stengel, M. & Spaldin, N. A. Carrier-mediated magnetoelectricity in complex oxide heterostructures. *Nat. Nanotechnol.* **3**, 46−50 (2008).
